# Global mapping of infectious disease

**DOI:** 10.1098/rstb.2012.0250

**Published:** 2013-03-19

**Authors:** Simon I. Hay, Katherine E. Battle, David M. Pigott, David L. Smith, Catherine L. Moyes, Samir Bhatt, John S. Brownstein, Nigel Collier, Monica F. Myers, Dylan B. George, Peter W. Gething

**Affiliations:** 1 Spatial Ecology and Epidemiology Group, Department of Zoology, University of Oxford, Oxford, UK; 2 Fogarty International Center, National Institutes of Health, Bethesda, MD, USA; 3 Department of Epidemiology and Malaria Research Institute, Johns Hopkins Bloomberg School of Public Health, Baltimore, MD, USA; 4 Department of Pediatrics, Harvard Medical School and Children's Hospital Informatics Program, Boston Children's Hospital, Boston, MA, USA; 5 National Institute of Informatics, Research Organization of Information and Systems, Tokyo, Japan

**Keywords:** surveillance, biosurveillance, cartography, public health, atlas, crowdsourcing

## Abstract

The primary aim of this review was to evaluate the state of knowledge of the geographical distribution of all infectious diseases of clinical significance to humans. A systematic review was conducted to enumerate cartographic progress, with respect to the data available for mapping and the methods currently applied. The results helped define the minimum information requirements for mapping infectious disease occurrence, and a quantitative framework for assessing the mapping opportunities for all infectious diseases. This revealed that of 355 infectious diseases identified, 174 (49%) have a strong rationale for mapping and of these only 7 (4%) had been comprehensively mapped. A variety of ambitions, such as the quantification of the global burden of infectious disease, international biosurveillance, assessing the likelihood of infectious disease outbreaks and exploring the propensity for infectious disease evolution and emergence, are limited by these omissions. An overview of the factors hindering progress in disease cartography is provided. It is argued that rapid improvement in the landscape of infectious diseases mapping can be made by embracing non-conventional data sources, automation of geo-positioning and mapping procedures enabled by machine learning and information technology, respectively, in addition to harnessing labour of the volunteer ‘cognitive surplus’ through crowdsourcing.

## 1. Introduction

The primary goal of this review is to establish the minimum set of information that is needed on the epidemiology of an infectious disease, to make an informed decision on the most appropriate techniques for mapping its global distribution. The assessment is intended to be applicable to all infectious diseases of clinical significance in humans, but makes no attempt to prioritize the case for mapping among the diseases considered.

More than 1400 species of infectious agents have been reported to cause disease in humans [1–3]. These include pathogens for some 347 diseases of sustained clinical importance, for which it is commercially viable to compile information relevant to their diagnosis, epidemiology and therapy, as a decision-support tool for clinicians [4,5]. Logistical constraints required a focus in this review on these clinically important diseases. Among these there are 110 diseases that pose a threat to non-immune travellers [4]. Sixty-two of these clinically significant diseases can be prevented by vaccination; 19 usually as routine childhood immunizations [4,6,7].

There are a variety of reasons for wanting to map the geographical distribution of an infectious disease. Mapping is a primary goal in spatial epidemiology [8–16]. Maps of disease distribution and intensity allow an immediate visualization of the extent and magnitude of the public health problem. When based on empirical evidence, maps can support carefully weighted assessments by decision makers on the advantages and disadvantages of alternative courses of action [17–19]. These may range from helping plan national scale intervention strategies [20,21] to advice for individuals on whether to vaccinate and/or provide prophylaxis before travel [6,22]. These maps can also document a baseline from which intervention success or failure can be monitored.

In addition, as modes of data gathering evolve and improve (for example, through enhanced electronic surveillance [17] and Internet-based health reporting [23], including HealthMap/ProMED [24,25], BioCaster [26,27] and Argus [28,29]) and techniques develop to exploit these data (for example, semi-automated rapid mapping), these geographical distributions (often referred to in this literature as baseline disease risk assessments) can also provide a ‘normal’ against which real-time outbreak alerts can be assessed for international biosurveillance [30–32].

Furthermore, as the portfolio of infectious disease distribution maps expands and their fidelity improves, the public health community will be better able to evaluate the factors that predispose a time and place to the origin [33,34], and emergence of infectious disease outbreaks [3,35–42]. Unfortunately, contemporary inferences about the fundamental ecology of infectious diseases (such as decreased species richness [43] and increased range size [44] with latitude and their potential for spread [45,46]) are crude spatially because they rely on data not systematically collected for this purpose and aggregated to the national level [4]. Ultimately, this improved basic understanding will help mitigate the processes that drive the diversity of infectious disease threats with which we contend [47].

There is, therefore, a clear need to perform baseline risk assessments for routine public health, improve biosurveillance and provide better long-term preparedness by improving fundamental epidemiological understanding [31].

An understanding of the public health benefit of the mapping of infectious disease is not new [48–50] and selected old examples for malaria include these references [51–55]. Historical disease cartography usually suffered at least one of the following problems. First, authors very rarely documented the evidence-base that was used to make the map. Second, when mapping was implemented before the advent of geographical information systems, significant errors arose simply as a function of cartographic skill. These errors were magnified enormously when working at global scales. Third, no assessment of the fidelity of the map or how this precision might vary spatially across the map extent was ever given. These limitations constrained significantly the public health utility of the maps and are to a greater or lesser extent resolved in many of the contemporary mapping efforts reviewed here.

Today, there are a range of different geographical distributions or baseline ‘risk’ maps available [56], which have been derived for a variety of purposes, by a wide community of public health cartographers using a diverse toolbox of mapping methods [8–16]. Moreover, the maps use a variety of disease-related metrics (occurrence, incidence, prevalence), and an even wider array of covariates to inform the predictions [8,57,58]. This complexity means that global comparisons between maps of different diseases are extremely difficult and wider synthesis remains elusive. In part, this review aims to help audit and navigate this diversity and the supplemental information provides an extensive bibliography arising from a systematic review of all diseases of clinical significance [4].

In this review, we also consider the minimum information requirements for disease mapping. When considering cartographic options for diseases of clinical importance, the first question is: do we know the life cycle of the pathogen, its vectors, reservoirs, hosts and routes of transmission? This sounds trivial, but for many pathogens there is still considerable uncertainty around the life history. Second, do we have information about the spatial and temporal patterns of the disease? Third, do we understand the dynamic processes of transmission that determine the patterns we observe in space and time? This level of detail will usually indicate some intimate epidemiological knowledge of covariates (temperature, rainfall, land use patterns, etc.), that can help in understanding the spatial and temporal distribution of a disease. Progression along this gradient of questions reflects increased basic epidemiological understanding and, therefore, an increased ability to map the disease. Fourth, it is important to know what quantity and quality of data are available for mapping. It is self-evident that more high quality contemporary data leads to more robust maps. Many obstacles exist that can make the relevant data scarce, however. For example, health-related data may be closely protected by governments and other institutions or these data may simply be scattered so widely in the formal literature that their systematic assembly is a significant logistical challenge. Fifth, it is also important to know whether previous credible mapping efforts have been conducted. This will help answer questions one through four and, broadly speaking, the longer the history of robust mapping activities, the increased likelihood of reliable mapping outcomes.

The ability to map a disease stems largely from the type of data that are available for mapping [10,15]. The accuracy of maps is then largely determined by the abundance, spatial representativeness and heterogeneity of those data [59]. Point data types used in disease mapping are generally geo-referenced occurrence or prevalence records. Occurrence data simply record an observation of a disease at a given location and time, and are characteristic of the data provided routinely by HealthMap/ProMED [24,25], BioCaster [26,27] and Argus [28,29]. The other commonly recorded point data are infection prevalence surveys, which not only locate a disease in time and space, but also measure the infected fraction of the sampled local population and thus, enable the standard quantification of the ‘abundance’ of a disease. This is often referred to as its endemicity [60]. An accurate global representation of the contemporary endemicity of a disease is a key achievement for infectious disease mapping, because it affords a rich diversity of operationally important public health inferences: for example, clinical burden [61,62] and basic reproductive number estimation [18,63] to inform national elimination feasibility assessment [20,64].

**Figure 1.**
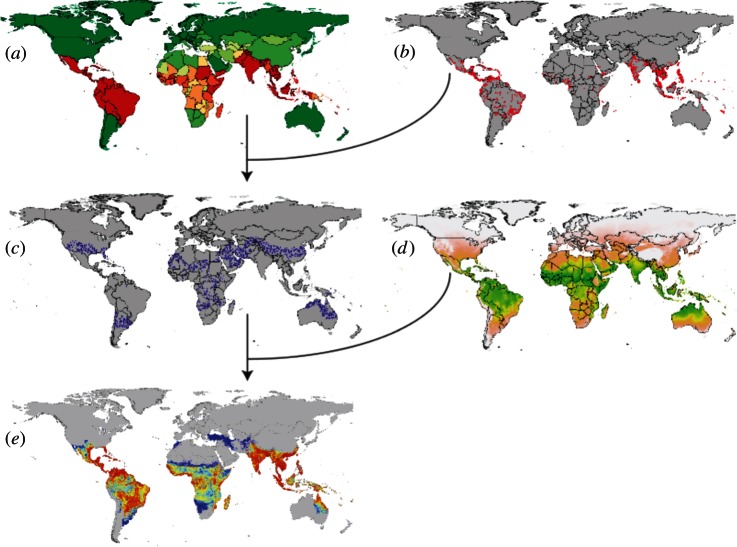
A schematic overview of a niche/occurrence mapping process (for example boosted regression trees (BRT)) that uses pseudo-absence data guided by expert opinion. Consensus based definitive extent layers of infectious disease occurrence at the national level (*a*) are combined with accurately geo-positioned occurrence (presence) locations (*b*) to generate pseudo-absence data (*c*). The presence (*b*) and pseudo-absence data (*c*) are then used in the BRT analyses, alongside a suite of environmental covariates (*d*) to predict the probability of occurrence of the target disease (*e*).

A wide range of approaches have been developed for empirical modelling of species and disease distributions, given data on point observations of occurrence [65], with the objective of identifying the fundamental niche of the target organism [66,67]. Of the plethora available, the boosted regression trees (BRT) method [68,69] is selected by the authors as a default for occurrence mapping. A schematic overview of the occurrence mapping process is provided in figure 1. This selection was based on a number of factors: first, in a review of 16 species modelling methods, BRT was one of the top performing methods evaluated using the area under the receiver operating characteristic curve (AUC) and correlation statistics [16,70]; second, the method is flexible in being able to accommodate different types of predictor variables (e.g. continuous or categorical data); third, it is easy to understand, implement and uses reliable, well documented and freely available R code [71]; and fourth, the resulting maps are simple to interpret and include a ranked list of environmental predictors. The authors also have extensive experience with this technique after a global scale project to map the distribution of the anophelines of public health importance [72–76]. These references provide a detailed statistical explanation and examples of how BRT was applied to species distribution mapping.

Model-based geostatistics (MBG) [77,78] has recently been more widely applied in infectious disease mapping [17,79–83] and is the technique of choice where data allow. There are several reasons for this. First, MBG deals explicitly with the spatial (and with extension temporal) autocorrelation of disease data; this is still widely ignored in occurrence mapping. Second, MBG models can be configured to offer a much more robust parameterization of factors that can affect disease endemicity (such as age of the individuals sampled, the diagnostic technique used, the influence of covariates etc.). Third, by fitting the models using Bayesian inference, outputs can be presented to show the full uncertainty of the prediction in all parts of the predicted maps. The main impediments to its wider use are the lack of bespoke software with which to implement the models and its relatively large computational burden.

We assume that advances with respect to occurrence mapping or MBG techniques may modify our guidance with regard to mapping techniques and elaborate on some of the generic improvements that may be made in infectious disease mapping in §4. Those we have favoured here are proved methods that can be applied now.

In summary, the objective of this review is to formalize the questions outlined in §1, in order to define rules for advocating specific cartographic techniques for a baseline risk assessment for each disease of clinical importance, and then to assess to what level this mapping potential has been realized. A substantial literature review has been conducted to collate the data required to make those cartographic suggestions evidence-based and is provided as electronic supplementary material.

## 2. Material and methods

### (a) Selection of infectious diseases of clinical importance

A total of 347 infectious diseases of clinical importance were selected for review based on the GIDEON database, accessed November 2010. GIDEON is an infectious disease information and diagnostic resource available online through subscription that derives its content from a range of sources including formal peer-reviewed journals and informal sources such as Ministry of Health reports [4,5]. This list was then revised to 355 diseases based on further re-definitions and decoupling of some groups. These diseases were placed into one of 11 classifications based on transmission type: animal contact, blood/body fluid contact, direct contact, endogenous, food/water-borne, respiratory, sexual contact, soil contact, unknown, vector-borne and water contact.

Revisions were as follows: mucosal and cutaneous leishmaniasis were re-classified as cutaneous/mucosal leishmaniasis, Old World and New World; the spotted fevers were also divided into New and Old World to better differentiate between the various species of bacteria and ticks that spread the disease in different parts of the world; malaria was split into *Plasmodium falciparum*, *Plasmodium vivax*, *Plasmodium ovale* and *Plasmodium malariae*, because variation in geographical range and epidemiological patterns of these pathogenic species would be masked if considered together; AIDS was removed and was combined with HIV; conjunctivitis-inclusion was similarly removed, and incorporated into trachoma; the umbrella term ‘adenovirus infection’ was divided into acute febrile respiratory disease (adenoviral), adenoviral haemorrhagic conjunctivitis, keratoconjunctivitis (adenoviral) and adenovirus infection; similarly, enterovirus infection was divided into enterovirus haemorrhagic conjunctivitis and enterovirus infection; human herpesvirus 6 was renamed Roseola; sandfly fever was added because of its possible impact on travellers; and avian influenza virus serotype H5N1 was added because of its epidemic potential.

### (b) Data assembly

#### (i) Natural history

Data were collected on the natural history of each infectious agent. Information on the genus and species, disease reservoir, vector species (if applicable), mode of transmission, incubation period, vaccine (where relevant) and geographical distribution was obtained using GIDEON. Taxonomic classifications were supplemented by the Tree of Life Project (http://tolweb.org). Further evidence regarding geographical distribution and vaccine development was found in the American Public Health Association's Control of Communicable Disease Manual [7].

#### (ii) Transmission dynamics

The basic reproduction number (*R*_0_) was used to quantify the transmission potential of the various aetiological agents. The *R*_0_ is defined as the average number of secondary infections produced when a single-infected individual is introduced into a fully susceptible population [84–87]. A literature search was conducted to obtain *R*_0_ values in humans and reservoirs of zoonotic diseases. The search was carried out in PubMed (http://www.pubmed.gov) using the terms ‘[disease name]’ and ‘reproduction number’ in the ‘all fields’ search box in September 2011. The search was then repeated replacing ‘reproduction number’ with ‘reproduction ratio’, ‘reproduction rate’, ‘reproductive number’, ‘reproductive ratio’ and ‘reproductive rate.’ That search pattern was reiterated with ‘[Genus species]’ or ‘[diseases synonym]’ replacing ‘[disease name],’ if applicable. This procedure was also performed in ISI-Web of Knowledge (http://isiwebofknowledge.com) in the ‘title/keywords/abstract’ field. These searches often produced few or no results and the entire search process would be conducted again using Google Scholar (http://scholar.google.co.uk). Data regarding *R*_0_ values and the reservoir species when relevant were abstracted from references obtained, and if multiple *R*_0_ estimates were reported among sources for a single disease, the range of estimates was recorded. The range for all *R*_0_ estimates was assumed to start from 0.

#### (iii) Thumbnail maps

To visualize the approximate endemic regions of a disease, simple maps were constructed from the distribution data provided by GIDEON. A list of 275 global countries and territories were coded as 1 for endemic and 0 for non-endemic for each listed disease. The database was then imported into ArcGIS 10 (ESRI 2010) and displayed as global maps at the national level.

#### (iv) Occurrence data availability and quality

To determine the relative amount of information available for the various infectious diseases, a search was done using only the disease name as the text term in PubMed on 4 November 2011 and using the species name in GenBank on 1 March 2012 (for selected diseases). Data on the number of feeds for each disease from the start of data collection were received from HealthMap and ProMED on 23 November 2011 and from BioCaster on 24 February 2012. Because only data from manual searches of PubMed has, to our knowledge, been used in mapping, we base our analyses on PubMed figures only, but provide the potential data from the other sources in the electronic supplementary material. These may improve the prospects for mapping of many of the diseases once the utility of these information sources has been confirmed by experiment.

### (c) Decision rules devised to categorize mapping options

**Figure 2.**
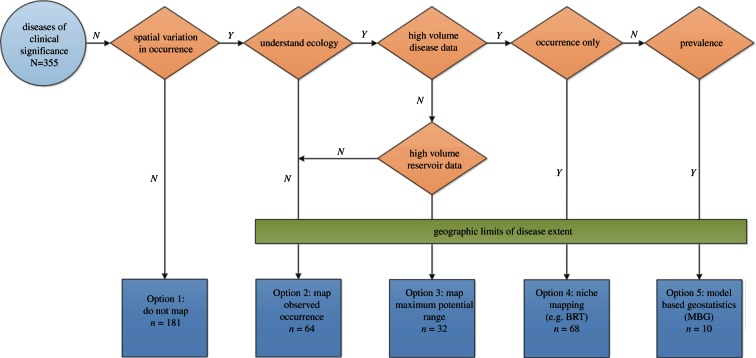
A schematic of the disease classification process. The classification system results in diseases being categorized into one of five options: (1) do not map; (2) map observed occurrence; (3) map maximum potential range of reservoir or vectors; (4) niche/occurrence mapping with BRT and (5) MGB-based endemicity maps.

Decision rules were created for disease mapping options, shown schematically in figure 2. The Option 1, *do not map*, classification was used for those conditions which are known to occur worldwide, and hence do not show sustained spatial variation in occurrence. The diseases within this category range from sexually transmitted diseases such as Chlamydia, viral agents such as Epstein–Barr Virus or rhinoviruses causing the common cold and endogenous diseases (infections caused by previously dormant or inapparent pathogens, often from the typical commensal microbial flora of humans—such as urinary tract infections caused by *Escherichia coli* or brain abscesses by *Staphylococcus aureus*). The incidence of these diseases may show enormous spatial variation. These differences are linked often to variation in human or human-related factors, however, and are best mapped using techniques associated with the cartography of non-infectious disease [88]. More traditional surveillance within this cosmopolitan distribution, therefore, may have a public health rationale and this is explored on a case by case basis in the electronic supplementary material. For most of these conditions, it would be useful to apply a simple mask of human population density to give a more realistic picture of where the disease is truly observed globally. Option 2, *map the observed occurrence*, would apply to diseases that have few data available and limited information regarding the disease ecology. A cut-off of fewer than 25 PubMed hits per endemic country was applied to designate a paucity of data for any operationally significant disease. For example, Mayaro virus has 90 search results on PubMed for 11 potentially endemic countries and, therefore, only about eight results per country. There has also not been a definitive reservoir host identified for Mayaro, which would be needed for the following option. Option 3, *map the maximum potential range*, is appropriate for a disease that also has fewer than 25 PubMed results per country, but information is available regarding reservoir or vector species that would place boundaries on the potential disease distribution, as is the case with African tick bite fever with its known vector distribution. Mapping of the disease using ecological niche modelling, Option 4, would implement *BRT technology on observed occurrence data*. Adequate information regarding occurrence of disease (greater than 25 PubMed hits per country) is needed to use this strategy. This information would be usefully supplemented with information on where the disease is not found, obtained through systematic searches or derived by expert opinion maps. If the authors were aware of systematic searches of occurrence data that were significantly richer than the PubMed hits, these were documented and the mapping option re-evaluated accordingly. Option 5, the *implementation of MBG to mapping*, is reserved for diseases that have more than 25 results per country of systematically recorded prevalence data. This strategy uses MBG for the creation of complete endemicity maps with detailed uncertainty metrics. The mapping option to be used is dependent on the amount and nature of the disease data available, implying that diseases currently classified for one option would be eligible for a higher grade in the future as further data become available.

### (d) Scoring the quality of existing mapping of the geographical distribution of disease

It was also of critical interest to obtain information regarding the extent to which the diseases had been previously mapped. A search was again conducted in PubMed using the text terms ‘[disease name/synonym]’ and ‘map’ as well as ‘[disease name/synonym]’ and ‘epidemiology,’ selecting for reviews in October 2011. If an excess of results were returned (more than 1000), this was further narrowed using the search terms ‘distribution’ or ‘global.’ For diseases transmitted by a specific vector, the search was repeated using the text terms ‘[vector species name]’ and ‘map.’ The same process was repeated for prominent reservoir species. The search was also performed using ISI-Web of Knowledge. Irrelevant references were removed from the search output, and all references regarding the spatial temporal distribution of a disease, vector or reservoir were checked to determine the parameter mapped (for example, occurrence, prevalence, incidence, or risk) and in what geographical region.

In order to allow for both relative and quantitative assessment of each map, we devised a metascore, which evaluated three criteria: data quality, geographical scope and the mapping technique used.

Data quality (out of nine) was scored in three ways. (i) Contemporariness, where three points were awarded if data less than 10 years old was used, two points for the use of data greater than or equal to 10 years to less than 20 years old, and one point for data greater than 20 years old. If no age could be identified, no points were given. For papers reporting a range of dates, the score was based on the most recent, with the exception of databases that provide country-specific estimates that were surveyed across different time periods. In that case, an additional half point (2.5) was given. (ii) Diagnostic accuracy, where three points were awarded for the use of data diagnosed by genotype or PCR, or in the case of vector maps, where advanced modelling techniques had been used on a large number of occurrence points. Two points were given to those studies that had used hospital or national health surveys or confirmed case reports; an additional half a point was gained if serological or immunological data had been used. Vectors maps received two points if simple interpolation techniques had been used on occurrence data. One point was awarded if cited literature had been used. One point was also given for unpublished health organization data collected as part of routine health management information systems (HMIS) or presumptive diagnosis, with a half point given to non-specific numerical data. The use of expert opinion in drawing vector maps was awarded one point. If the data came from an unknown source, or was not listed in the article, no points were awarded. (iii) Geo-positional accuracy, where three points were awarded for the use of data coupled with GPS coordinates, two points if survey coordinates could be derived from supporting maps, or data was provided to administrative level 1; an additional half a point was earned if administrative level 2 was used, or towns and villages were specified. One point was gained if approximate coordinates of unknown provenance or country level data was present. Expert opinion ranges obtained from cited literature received half a point. If no geo-positional data was associated with the map, no points were awarded.

The geographical scope was scored out of 100. The GIDEON endemic country lists for each disease were converted into national populations at risk using the UN population data from 2010 [89]. Each map was assessed for how many countries were included (rounded up to the national level, to match the resolution of GIDEON), and population covered was calculated and expressed as a percentage (out of 100%) of the GIDEON endemic total.

The mapping technique used (mapping option used/theoretically best mapping option) was calculated using the criteria outlined above, each map was evaluated for the mapping option used (for example, if BRT modelling techniques had been used, the map was to Option 4 standard), and was related to the potential mapping option that could be used, based upon the amount and quality of data present for that disease. For instance, if a map of Lassa fever (which is an Option 4 disease owing to there being more than 25 PubMed hits per country) only uses occurrence points (Option 2 standard), a score of 2/4 would be achieved.

The metascore was then calculated as the product of these figures ([Quality]/9 × [Scope] × [Option Used]/[Option Potential]) resulting in a maximum of 100. Scores of greater than or equal to 75 per cent were deemed to have evaluated the global distribution of the specific disease to a satisfactory standard.

## 3. Results

The electronic supplementary material provides full details of all the epidemiological and mapping evidence collated and scored and the decision rules applied. The electronic supplementary material includes a summary page on each of the 355 diseases with details of the natural history, transmission, quantity of data available, quality of data from previously published maps and recommendations for future mapping endeavours. The information included on natural history was the ICD-10 code, transmission classification (table 1), type of pathogen (agent), taxonomic details, mode of transmission, reservoir species (host organism that is a source of infection or potential reinfection of humans) and incubation period.

**Table 1.** The number of clinically important infectious diseases and the subset of those with a rationale for mapping by transmission category (see §2).

classification	clinically significant diseases (*n* = 355)	diseases with rationale for mapping (*n* = 174)
animal contact	20	9
blood/body fluid contact	14	5
direct contact	23	7
endogenous^a^	35	0
food/water-borne	82	36
respiratory	39	9
sexual contact	11	2
soil contact	21	14
unknown	11	4
vector-borne	88	80
water contact	11	8

^a^Endogenous infections are those caused by previously inapparent or dormant pathogens arising from the typical commensal microbial flora of humans.

The epidemiological characteristics highlighted include the vaccine availability, and estimates of the basic reproduction number (*R*_0_) in human and reservoir populations, where applicable. A number of diseases (126) were considered to have an *R*_0_ value of less than 1 because they are primarily zoonotic diseases. Citations were provided to support that transmission occurs mainly in animals. The *R*_0_ estimates ranged from point source outbreaks of diarrhoeal diseases or less than 1 for zoonoses to 100 for *P. vivax* malaria and Ross River virus and 1000 for *P. falciparum* malaria. Estimates were not obtained for many of the reservoir species, but for those that were found, the range was from 1.06 for Old World mucocutaneous leishmaniasis in dogs to 28 for West Nile fever virus in birds.

Occurrence details included information on the number of PubMed and GenBank hits, relevant reports from HealthMap, ProMED and BioCaster feeds, and the approximate number of endemic countries. A table of previously published maps was included incorporating information on whether the map is of the disease, vector or host reservoir; geographical scope; data quality score; mapping option used; metascore; citation.

The option for future mapping (figure 2) was determined using the PubMed hits returned and the number of endemic countries per diseases (see the electronic supplementary material). A total of 181/355 were classified as Option 1 (do not map); 64 were classified as Option 2 (map observed occurrence); 32 were classified as Option 3 (map maximum potential range); 68 were classified as Option 4 (map using BRT) and 10 were classified as Option 5 (map using MBG).

There are trends within the diseases that have a strong rationale for mapping. Unsurprisingly, endogenous diseases exhibit little sustained spatial variation in occurrence, whereas those transmission categories that are inherently linked to some feature of the environment, or other factor that varies on a global scale, such as vector-borne disease, water contact and soil contact tend to show greater variation. The remaining transmission types have just under half of the diseases showing differing global patterns of distribution. Similar trends are also apparent when we consider the occurrence of agents of disease—nearly two-thirds of diseases caused by parasites show tendency to vary over a spatial scale, as do 61 per cent of all viruses; on the other hand, there is evidence for spatially variable distributions in only 28 per cent of bacteria. Clearly, these sets of results are inherently linked; of the 61 viral diseases that would benefit from having mapped distributions, 41 are vector-borne and a further eight are soil contact; of those bacterial species that are not endemic worldwide, about two-thirds are vector-borne. Such a trend is not so apparent when considering parasitic diseases and their routes of transmission (many are food/water-borne). This could be due to their requirements for external development, and thus potentially environmentally determined life cycles.

**Figure 3.**
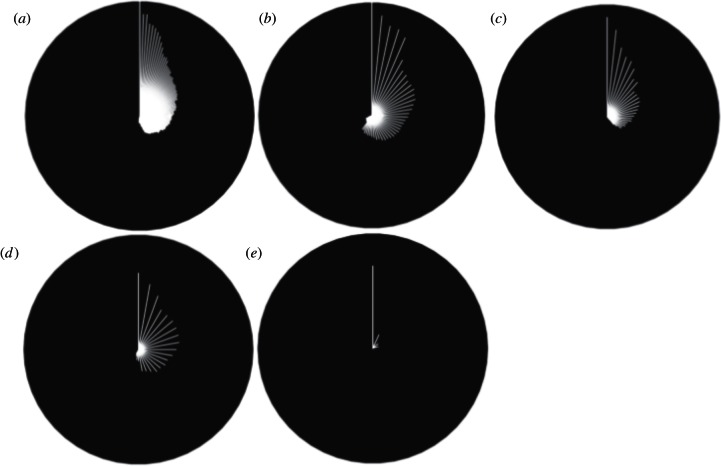
Radial plots for all diseases with a rationale for mapping, ordered clockwise, by metascore (white line). A white line from the centre to the edge of the circle would show a perfect metascore. (*a*) Reflects all diseases (*n* = 174 of 355), (*b*) viral diseases (*n* = 62 of 101), (*c*) parasitic diseases (*n* = 61 of 96), (*d*) bacterial diseases (*n* = 36 of 128), and (*e*) comprises fungal (*n* = 9 of 17), protoctistan (*n* = 2 of 2) and diseases of unknown pathogen (*n* = 4 of 10). Note that there was one algal disease, which did not have a rationale for mapping and is not shown in this diagram.

Of the 174 diseases with strong rationale for mapping, only seven had maps that scored higher or equal to 75 per cent on the metascore. These were coltiviruses (Old World), dengue, Lassa fever, Mayaro, monkey pox, *P. falciparum* and *P. vivax*; all vector-borne diseases. Figure 3*a* shows radial plots of all the 174 diseases with a rationale for mapping, as well as separate plots by agent (figure 3*b*–*e*). The white line represents the highest scoring metascore for each disease; the black space above each individual line equates to the information deficit present.

## 4. Discussion

We have collated a significant amount of information on 355 diseases of clinical importance and have made evidence-based suggestions on the appropriate cartographic approaches to use in mapping each disease. These have been summarized in the results and are elaborated for each disease in the electronic supplementary material. In the following sections, we review some of the common omissions in existing maps and look to novel data sources, new techniques and information technology developments that may change the future landscape of infectious disease mapping.

This review has provided the opportunity to make some preliminary observations on some of the common omissions in infectious disease mapping that might be considered when embarking on new cartographies. They are as follows.

### (a) Other relevant maps

The most consistent omission is the lack of additional information that can provide significant epidemiological insight—often referred to as ‘expert opinion’. These definitive extent data can be an ad hoc collection for each disease that may include information on biological and biogeographic limits (often as range maps), as well as, further distribution or occurrence data on intermediate and reservoir hosts. There are several occurrence mapping methods that can use this information, such as weighted forms of BRT that have been trialled extensively with respect to the anophelines [72–75] (figure 1). They do this by overcoming the biogeographic and taxonomic ignorance of all occurrence mapping techniques that assume the globally realized niche approximates the fundamental niche. The careful use of definitive extent data would substantially reduce the degree to which inferences are required.

### (b) Formalizing expert opinion

Further investigation is also advised on using the Cooke method to help determine the importance ascribed to the expert opinion [90,91]. Essentially these methods allow a simple way to gauge the accuracy of an expert source by testing their knowledge on a set of subject related questions to which the answers are well known. For a cartographic problem set, this could be very easily formalized by rating answers for a related disease we know the distribution of extremely well. It may be possible to link this with BRT and formalize the weights that are ascribed to other relevant epidemiological information.

### (c) Human population distribution

There is a systematic deficit in the use of human population distribution maps [92,93], both as a mapping covariate and for determining the population at risk of infection or the reservoir of infection. Some effort may also be invested in incorporating the latest human population surfaces into the information suite. The diseases for which human population distribution may help refine risk assessments, including both those with a rationale for mapping and those ubiquitous clinically important diseases for which the recommendation was not to map, have been highlighted (see the electronic supplementary material).

### (d) Refining of environmental covariates

Most cartographic applications use environmental covariates crudely without any adjustment to the epidemiology of the diseases concerned. Where detailed information and experiments on the environmental responses of a disease have been conducted it has proved valuable to combine this with the covariate. An example would be the way that temperature data have been used not only to map the environmental limits of *P. falciparum* and *P. vivax* globally [94], but have also been transformed into indexes of transmission suitability. These indexes were more strongly selected for by the model than untransformed covariates in endemicity mapping. The diseases to which such advances may be relevant are indicated (see the electronic supplementary material).

### (e) Public health interventions

It is still rare for geographically specific intelligence on public health interventions to be used in the mapping of diseases. Such information could be used in the same way as other ‘expert opinion’ data sources by BRT. Where human interventions have significantly affected the distribution of a disease, for example vaccine coverage in a population [95–97], this has been identified. We have sought to identify those diseases for which this information may be relevant but have not searched systematically for the availability of relevant public health information.

There are many potential novel data sources that may be used for global infectious disease mapping. The resources described below have never been used systematically to address the paucity in occurrence data across the range of infectious diseases reviewed. Substantial progress will be made from exploiting the geospatial information in the formal literature (e.g. PubMed, www.ncbi.nlm.nih.gov/pubmed) and in genetic and protein sequence databases (e.g. GenBank, www.ncbi.nlm.nih.gov/genbank). The potential information available has been identified for each disease in the electronic supplementary material and is further summarized in table 2.

**Table 2.** The cartographically relevant holdings of the National Center for Biotechnology Information PubMed and GenBank systems. The searches were conducted on 4 November 2011 and 1 March 2012, respectively.

system	PubMed	GenBank
start year	1946 [98]	1982 [99]
frequency of updates	daily [98]	Daily [100]
number of species catalogued	>250 000 [100]	>250 000 [100]
approximate number of entries	21 million [101]	340 million [100]
number of clinically relevant diseases for which data are available	168	155
occurrence point sources for mapping	526 564	672 327

Significant prospects for the rapid acquisition of occurrence data are also clearly possible from online outbreak alert resources (i.e. HealthMap/ProMED [24,25], BioCaster [26,27] and Argus [28,29] records). The potential information available has been identified for each disease in the electronic supplementary material and is further summarized in table 3 for those systems where data can be freely shared.

**Table 3.** Geo-positioned occurrence data archived by the HealthMap and BioCaster online disease outbreak reporting systems. HealthMap uses automated text processing to classify and position alerts that are then confirmed by a human analyst [25]. BioCaster has automated text processing to classify and position alerts processed through a multilingual ontology [26]. The totals were assembled using data provided for HealthMap on 23 November 2011 and BioCaster on 24 February 2012.

system	HealthMap	BioCaster
start year	2006	2006
approximate posts per day	300 [24]	100 [29]
number of languages	10 (J. S. Brownstein 2012, personal communication)	11 [102]
number of diseases tagged	245 (J. S. Brownstein 2011, personal communication)	230 (N. Collier 2012, personal communication)
number of clinically relevant diseases for which data are available	84 of 245	99 of 230 (N. Collier 2012, personal communication)
total occurrence points	337 105 (J. S. Brownstein 2011, personal communication)	189 361 (N. Collier 2012, personal communication)
occurrence point sources for mapping	66 284 (J. S. Brownstein 2011, personal communication)	140 038 (N. Collier 2012, personal communication)

Finally, there is a revolution occurring in both the volume and public availability of data about the health and wellbeing of individuals and populations through various forms of social media [103]; most notably Twitter (twitter.com). This is an online social media site that allows users to post ‘Tweets’; messages less than or equal to 140 characters which are freely available to all. It took 3 years to reach the first billion Tweets, but by March 2011, it took only a week to reach one billion posts and 140 million Tweets are now posted daily with an increasing number of them automatically geo-positioned. This wealth of accurately geo-positioned information has already begun to be harvested for public health purposes. Twitter feeds surrounding the 2009 H1N1 flu outbreak were analysed and found to predict outbreaks one to two weeks in advance of traditional surveillance [104,105]. Tweets can also be analysed to identify a broader range of health-related terms such as symptoms, syndromes and treatments to illuminate geographical patterns in syndrome surveillance [106].

Our optimism about the future use of social media is tempered by the realization that the main contemporary issue in disease mapping, of dealing with the lack of relevant data, will subside, and that our new challenges will be informatics, developing systems and processes to take on the big data challenges of the future. This is discussed in the following section.

There are also many novel techniques that may be used to improve the prospects of global infectious disease mapping, notably automation through machine learning and harnessing the cognitive surplus. In the defined schema (figure 2), it is more logistically and technically difficult (and thus expensive) to map diseases from Option 1 (do not map) through to Option 5 (map endemicity with MBG). It is also more expensive to deal with conditions for which data retrieval is a significant logistical obstacle. This will be directly proportional to the number of PubMed and other (see earlier) data source hits identified.

The HealthMap and BioCaster systems have pioneered machine learning algorithms that automatically classify relevant reports, identify the infectious disease of interest and determine the geographical location of the outbreak. Scaling these to cope with this potential data deluge is a non-trivial but largely technical problem. Ideally, the results of this process should be audited and verified by subject matter experts but this is non-scalable, time consuming and prohibitively expensive.

As an alternative, developments in social computing have led to increased interest in using large numbers of non-experts as a cheaper and scalable method for data filtering: the so-called crowdsourcing or distributed cognition [107,108]. Currently established ways to crowdsource exist (i) framing filtering tasks as fun online games, incentivizing users to filter data for free [109] and (ii) posting the task online and seeking non-experts using a pay-per-example setting as pioneered by the Amazon Mechanical Turk system [110,111]. The central idea is that, if questions can be structured in a simple and intuitive way, and presented to a large number of individuals, the central tendency of responses is likely to provide an accurate answer. Crowdsourcing is particularly appealing in the context of filtering social media disease reports because of the non-expert nature of key components of the task, such as geo-positioning. Crowdsourcing is not, of course, a panacea for data filtering. The reliability of contributors must be quantitatively assessed and iteratively adjusted for, again with reference to a gold-standard reference set of externally validated results.

In conclusion, this systematic review has shown that we have an astonishingly poor knowledge of the global distribution of the vast majority of infectious diseases of clinical importance. Less than 5 per cent of clinically important infectious diseases have been mapped reliably. This presents clear obstacles to advances in determining the global burden of these conditions, our ability to differentiate outbreaks of concern in international biosurveillance, and our ability to understand the geographical determinants of disease emergence, past, present and future. We have shown that contemporary solutions exist to enable us to use new data and new technology to rapidly improve the cartography of a wide range of clinically important pathogens. Few conceptual barriers exist to making rapid progress and to ‘seeing further’ into the relatively unknown landscape of infectious disease mapping.
